# The Activation of the Microglial NLRP3 Inflammasome Is Involved in Tuberous Sclerosis Complex-Related Neuroinflammation

**DOI:** 10.3390/ijms26157244

**Published:** 2025-07-26

**Authors:** Ran Ding, Shengxuan Zhang, Linxue Meng, Lingman Wang, Ziyao Han, Jianxiong Gui, Jiaxin Yang, Li Cheng, Lingling Xie, Li Jiang

**Affiliations:** 1 National Clinical Research Center for Child Health and Disorders, Chongqing 400014, China; 2 Department of Neurology, Children’s Hospital of Chongqing Medical University, Chongqing 400014, China; 3 Ministry of Education Key Laboratory of Child Development and Disorders, Chongqing 400014, China; 4 Chongqing Key Laboratory of Child Neurodevelopment and Cognitive Disorders, Chongqing 400014, China

**Keywords:** tuberous sclerosis complex, cortical tubers, microglia, NLRP3 inflammasome, IL-1β

## Abstract

Tuberous sclerosis complex (TSC) is a systemic disease caused by mutations in either the TSC1 (encoding hamartin) or TSC2 (encoding tuberin) gene, with mutations in the TSC2 gene potentially leading to more severe clinical symptoms. Neurological symptoms are a common clinical manifestation of TSC, and neuroinflammation is thought to play an important role. Glial cells are a major source of neuroinflammation, but whether microglia are involved in the activation of the NOD-like receptor protein 3 (NLRP3) inflammasome and the expression of interleukin-1β (IL-1β) in TSC patients remains unclear. We used a transcriptome sequencing dataset for bioinformatics analysis to explore the differences in the expression of microglial inflammasome-associated hub genes. TSC2 knockdown (TSC2 KD) microglia (HMC3 cell line) were generated by lentivirus, and the expression of inflammasome-associated hub genes, microglial activation, and NLRP3 inflammasome activation were verified. In addition, experiments were performed to explore the regulatory effects of rapamycin. Bioinformatics analysis identified a total of eight inflammasome-associated hub genes. By detecting GFP fluorescence, TSC2 mRNA, TSC2 protein expression, and the phosphorylation of the mammalian target of rapamycin (p-mTOR)/mTOR, we confirmed that the TSC2 KD microglia model was successfully established. Compared with the control group, the TSC2 KD group presented higher mRNA levels and fluorescence intensities of microglia AIF1 and CD68, as well as greater reactive oxygen species (ROS) production. Eight inflammasome-associated hub gene mRNA assays revealed that the expression of the NLRP3 and IL1B genes was increased. Compared with the control group, the TSC2 KD group presented increased levels of NLRP3 and Pro-IL-1β proteins in cells and Cleaved-Caspase 1 and Cleaved-IL-1β proteins in the supernatant, suggesting NLRP3 inflammasome activation. Rapamycin intervention alleviated these changes, demonstrating that the TSC2 gene regulation of microglial activation and NLRP3 inflammasome activation are correlated with mTOR phosphorylation. In conclusion, microglia are activated in TSC patients and participate in the NLRP3 inflammasome-associated neuroinflammatory response, and rapamycin treatment can alleviate these changes.

## 1. Introduction

Tuberous sclerosis complex (TSC) is a rare disease with systemic involvement, resulting from mutations in the TSC1 or TSC2 genes, with an incidence of approximately 1/6000–10,000 births [1]. The proteins encoded by the TSC1 and TSC2 genes bind to carry out their biological functions, but mutations in the TSC2 gene are linked to increased pathogenicity and more severe clinical symptoms than those in TSC1 [2]. Mutations in the TSC1/2 genes lead to the phosphorylation of the downstream molecule mammalian target of rapamycin (mTOR) [3]. The dysregulation of the mTOR signaling pathway results in atypical development of the cerebral cortex, affecting cortical stratification, cell volume, and axon and dendrite growth [4]. The majority of TSC patients suffer from neurologic hamartoma, a tumor-like malformation caused by the misassembly and misalignment of normal tissues during development, with a prevalence of cortical tubers as high as 90%. Autopsy reports reveal that cortical tubers are filled with numerous giant cells, dysmorphic neurons, activated astrocytes, and microglia, which contribute to focal neurological abnormalities. Cortical tubers are implicated in drug-resistant epilepsy and other neurological symptoms, and surgical resection has shown promise in reducing seizures, suggesting potential benefits in targeting tubers [5].

Several studies have demonstrated significant neuroinflammation in the brain tissue of TSC patients and that the cytokine interleukin-1β (IL-1β) is involved in the TSC neuroinflammatory response [6,7,8,9]. However, more studies have focused on astrocytes, and studies of microglia are limited [10,11]. Kagitani-Shimono et al. [12] detected significant microglial activation in the brains of TSC patients with refractory epilepsy using functional magnetic resonance imaging. Boer et al. [13] first identified IL-1β from microglia in human cortical tubers, but the level of IL-1β did not increase in mouse microglia when the astrocytic Tsc1 gene was conditionally knocked out [10]. Thus, it remains unclear whether TSC microglia independently produce excess IL-1β. Previous studies have shown that promoter-specific hypomethylation and TLR4/IL-1R are associated with IL-1β overexpression in TSC patients [14,15,16]. However, the classical activation pathway of IL-1β, i.e., the inflammasome-associated signaling pathway, remains unexplored. The inflammasome is the upstream molecule of IL-1β and includes various types, such as NOD-like receptor protein 3 (NLRP3). NLRP3 activates the inflammasome by binding to apoptosis-associated speck-like protein containing a CARD (ASC) and precursor cysteinyl aspartate-specific proteinase 1 (Pro-Caspase 1), producing the biologically active Cleaved-Caspase 1. Cleaved-Caspase 1 is known to generate Cleaved-IL-1β by cleaving Pro-IL-1β, which subsequently leads to inflammatory injury [17]. In many neuroinflammatory diseases, microglia regulate inflammation and activate the inflammasome [18,19]. However, whether the TSC microglial inflammasome is abnormally activated remains unknown. Therefore, probing the activation of the microglial NLRP3 inflammasome and the production of Cleaved-IL-1β could help us to further understand the mechanism of TSC neuroinflammation.

The mTORC1 inhibitor rapamycin was first isolated from *Streptomyces hygroscopicum* in the soil of Easter Island and has been used as a targeted therapeutic agent for the treatment of TSC [20]. Several previous studies have shown that NLRP3 inhibitors reduce the extent of injury, whereas rapamycin results in similar interventions [21,22,23]. Autophagy may alleviate inflammation by inhibiting the production of mitochondrial reactive oxygen species (ROS), degrading the assembled inflammasome, and isolating Caspase 1 from contact with Pro-IL-1β. When mTORC1 activation results in impaired autophagy, NLRP3 inflammasome activation is significantly increased. The autophagy inducer rapamycin has been shown in several studies to inhibit microglial NLRP3 activation and IL-1β production [24,25]. Thus, rapamycin is a potential modulator of neuroinflammation in TSC microglia, but experiments are needed to verify this hypothesis.

Sequencing technology and omics analysis methods enable more precise target identification. However, only a limited number of studies have sequenced cortical tubers. Therefore, in this study, we chose the EGAS00001002485 dataset of RNA-seq data from the cortical tubers of TSC patients for secondary analysis. Cellular experiments were used for further validation via bioinformatics analysis, and the activation of microglia and the NLRP3 inflammasome was investigated. In addition, the modulatory effect of rapamycin on TSC2 KD microglia was explored in this study.

## 2. Results

### 2.1. General Exploration of the Dataset

**Figure 1:**
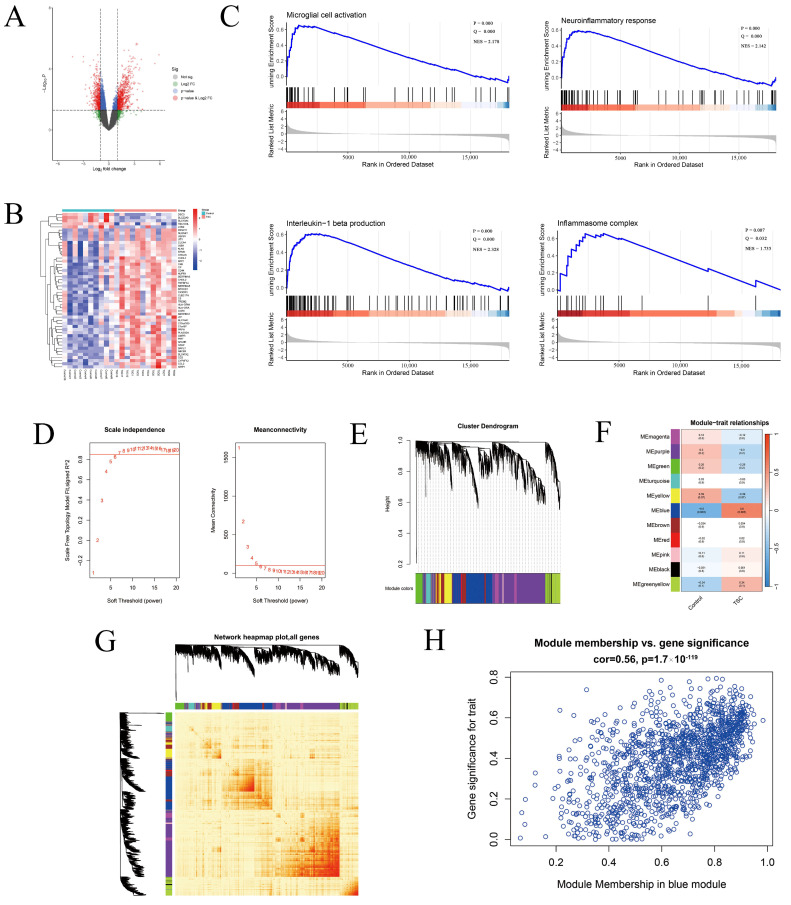
General exploration, GSEA, and WGCNA of the EGAS00001002485 dataset. (**A**) The volcano map shows the changes in mRNA expression in cortical tubers, and the red dots represent the differentially expressed genes. (**B**) Heatmap showing the top 50 differentially expressed genes, with red representing high expression and blue representing low expression. (**C**) GSEA revealed that pathways related to the neuroinflammatory response, microglial cell activation, the inflammasome complex, and IL-1β production were significantly enriched. (**D**) R^2^ was set to 0.85 (left red line), and the function was used to automatically filter 7 as the optimal soft threshold. The red line in the image on the right represents the mean connectivity when the soft threshold is 7. (**E**) Clustering tree and module delineation of co-expressed gene modules of TSC. (**F**) The module–trait relationships of the 11 gene modules associated with TSC were analyzed, and the blue module presented the highest correlation with TSC. (**G**) The topological matrix heatmap of the modular gene co-expression network shows high intermodule differentiation and high intramodule gene correlation. (**H**) The scatter plot revealed that genes in the blue module were positively correlated with TSC (cor = 0.56, *p* = 1.7 × 10^−119^).

A total of 1234 differentially expressed genes (DEGs) were identified, including 513 downregulated genes and 721 upregulated genes (Figure 1A). The heatmap shows the expression of the fifty DEGs with the highest |log fold change (FC)| values, with the majority of genes in the TSC group showing upregulated expression (Figure 1B).

### 2.2. Gene Set Enrichment Analysis

In addition to the microglial activation pathway [normalized enrichment score (NES) = 2.178, Q value = 0.000, *p* value = 0.000], the neuroinflammatory pathway (NES = 2.142, Q value = 0.000, *p* value = 0.000), the IL-1β production pathway (NES = 2.328, Q value = 0.000, *p* value = 0.000), and the inflammasome complex pathway (NES = 1.735, Q value = 0.032, *p* value = 0.007) were significantly enriched (Figure 1C).

### 2.3. Weighted Gene Co-Expression Network Analysis

In the Weighted gene co-expression network analysis (WGCNA), when the soft threshold power β is set to 7, the scale-free topology fitting index R^2^ reaches 0.85 (Figure 1D). The hierarchical cluster tree illustrates the different modules, represented by different colors, which are ultimately combined into 11 modules (Figure 1E). The network heatmap of all genes demonstrates that the modules are well differentiated from each other and that the genes within the modules are highly correlated (Figure 1G). The module–trait relationship plot shows that the blue module, which contains 1438 genes, is most strongly associated with TSC, suggesting its potential significance in TSC-related biological processes (cor = 0.56, *p* < 1.7 × 10^−119^; Figure 1F,H).

### 2.4. Functional Analysis and Inflammasome-Related Hub Gene Screening

**Figure 2:**
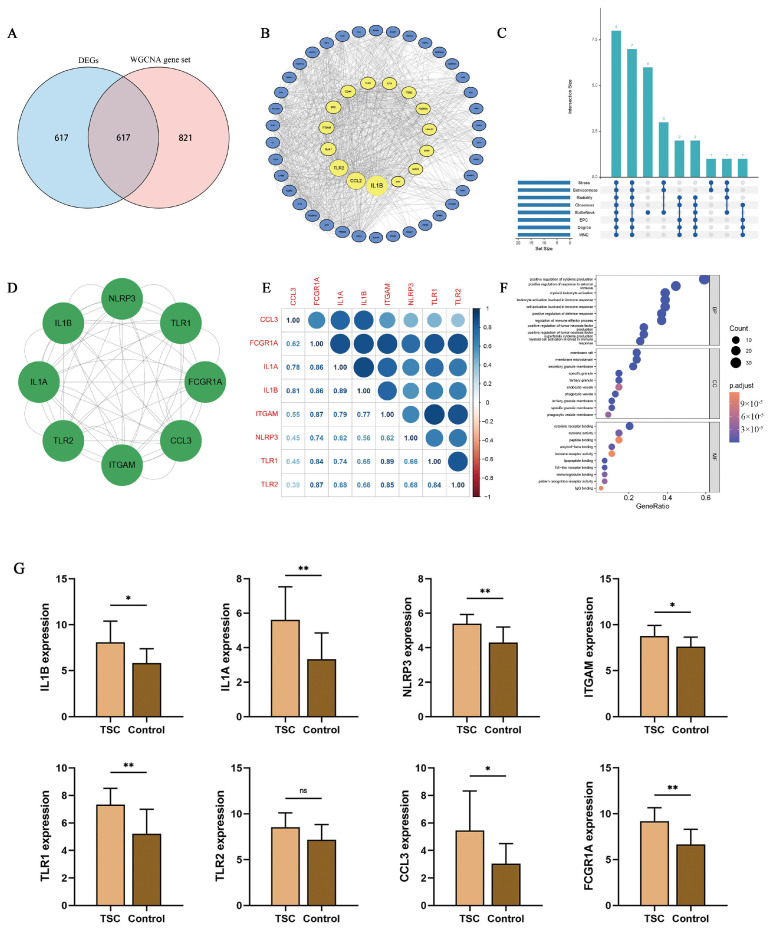
Identification of inflammasome-related hub genes. (**A**) Venn diagram showing 617 intersecting genes. (**B**) PPI network analysis reveals interactions between proteins encoded by 53 GAMNIs. (**C**) An UpSet diagram demonstrating the specifics of 8 algorithms of CytoHubba for screening inflammasome-related hub genes, and 8 genes were screened. Each dot represents a certain algorithm to its left, and the numbers on the bar graph represent the number of genes selected by each algorithm below it. (**D**) PPI network analysis revealed interactions between proteins encoded by inflammasome-related hub genes. (**E**) Correlation analysis between inflammasome-related hub genes revealed that these genes were positively correlated. (**F**) GO enrichment analysis of inflammasome-related hub genes. (**G**) Significance analysis of differences in inflammasome-related hub genes. ns *p* > 0.05, * *p* < 0.05, ** *p* < 0.01.

The Venn diagram shows the intersection of the DEGs and WGCNA gene sets, with 617 genes included in the intersection (Figure 2A). For the microglia gene set and the inflammasome gene set, 54 genes associated with the microglial NLRP3 inflammasome (GAMNIs) among the intersecting genes were selected. The protein–protein interaction (PPI) network suggested complex interactions among 53 proteins (Figure 2B, Supplementary Table S3). To prioritize the most critical genes, we employed each of the eight algorithms in the Cytoscape software version 3.9.0 to score genes and extracted the genes selected by all eight algorithms as inflammasome-related hub genes. An UpSet plot was generated to visualize the screening results, pinpointing eight inflammasome-related hub genes: IL1B, IL1A, NLRP3, TLR1, TLR2, ITGAM, CCL3, and FCGR1A (Figure 2C). The PPI network revealed strong connections among these genes (Figure 2D). Correlation analysis demonstrated positive correlations among all eight genes (Figure 2E). These genes were subjected to Gene Ontology (GO) functional enrichment analysis. The Biological Process (BP) program includes functions such as the positive regulation of cytokine production, glial cell activation, and the neuroinflammatory response; the Cellular Component (CC) is involved in the inflammasome complex; and the Molecular Function (MF) involves cytokine activity and IL-1β receptor binding (Figure 2F). These findings suggest that inflammasome-related hub genes may be involved in glial cell activation and inflammasome generation, which in turn mediate increased inflammatory factor production. A comparison of inflammasome-related hub gene expression between the two groups revealed that the expressions of IL1B, IL1A, NLRP3, TLR1, ITGAM, CCL3, and FCGR1A were significantly greater in the TSC group than in the control group, whereas no significant difference in TLR2 expression was detected between the two groups (Figure 2G).

### 2.5. Establishment of a TSC2 Knockdown Microglial Model

**Figure 3:**
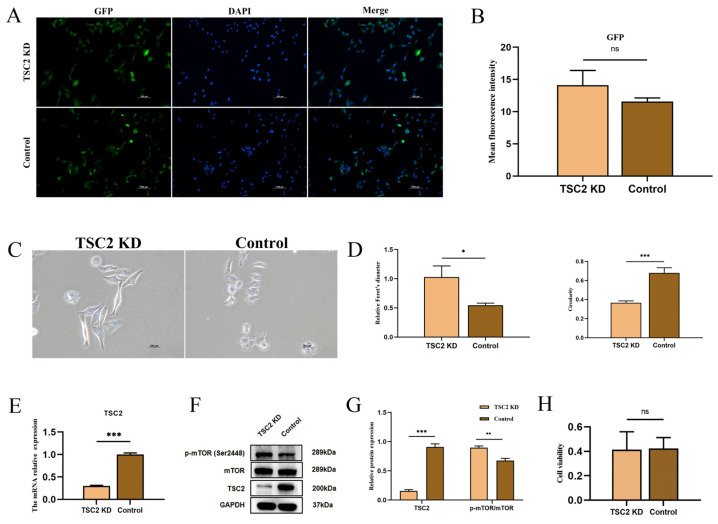
TSC2 KD microglia model construction. (**A**) HMC3 cells expressed GFP fluorescence after transfection with lentivirus. (**B**) Comparison of the GFP fluorescence intensity between the two groups. (**C**) Morphology of microglia after lentiviral transduction. (**D**) TSC2 KD cells have increased Feret’s diameter and decreased circularity. (**E**) The mRNA level of TSC2 was reduced in the TSC2 KD group. (**F**,**G**) The protein level of TSC2 and the phosphorylation of mTOR were detected by Western blot. Compared with control microglia, TSC2 KD microglia presented significantly lower TSC2 protein expression and significantly higher phosphorylation levels of mTOR. (**H**) A CCK-8 assay revealed no significant difference in microglial activity between the two groups. N = 3; ns *p* > 0.05, * *p* < 0.05, ** *p* < 0.01, *** *p* < 0.001.

After lentiviral transduction and the selection of a stable cell line, all human microglial cells (HMC3) expressed GFP fluorescence, and the fluorescence intensity was similar in both groups, suggesting the successful transcription of the vector inside the cells (Figure 3A,B). After lentiviral transfection, the morphology of microglia in the TSC2 KD group was altered. Naked-eye observation revealed that the cells in the TSC2 KD group were more spindle-shaped (Figure 3C). Morphological measurements revealed that, compared with those in the control group, the Feret’s diameter of the cells in the TSC2 KD group was greater, and the circularity was lower (Figure 3D). Furthermore, the Real-time quantitative polymerase chain reaction (RT-qPCR) revealed a significant decrease in the expression of the TSC2 gene in the TSC2 KD group, and Western blotting further demonstrated the downregulation of the TSC2 protein (Figure 3E–G). The phosphorylation level of the S2448 site of the downstream protein, mTOR, was increased (Figure 3F,G). These findings indicate that the model was successfully established. The results of the CCK-8 experiment revealed no significant difference in absorbance between the two groups, suggesting that the downregulation of TSC2 does not affect microglial activity (Figure 3H).

### 2.6. Microglial Activation Assay

**Figure 4:**
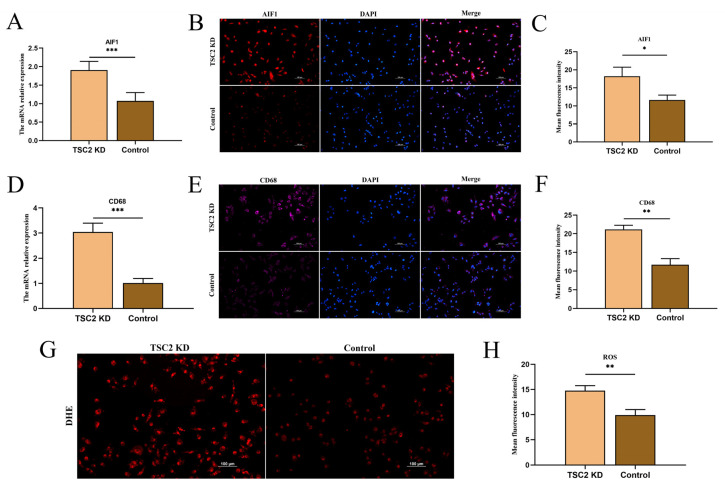
Detection of HMC3 cell activation and ROS levels. (**A**) The mRNA level of AIF1 was elevated in the TSC2 KD group. (**B**,**C**) AIF1 fluorescence intensity was greater in TSC2 KD HMC3 cells than in control HMC3 cells. (**D**) The mRNA level of CD68 was elevated in the TSC2 KD group. (**E**,**F**) The CD68 fluorescence intensity of TSC2 KD HMC3 cells was greater than that of control cells. (**G**,**H**) The intensity of ROS fluorescence in TSC2 KD HMC3 cells was greater than that in control cells. DHE: Dihydroethidium; N = 3; * *p* < 0.05, ** *p* < 0.01, *** *p* < 0.001.

We assessed the mRNA levels of the AIF1 and CD68 genes by RT-qPCR. The results revealed that the AIF1 and CD68 mRNA levels in TSC2 KD cells were significantly greater than those in control cells (Figure 4A,D). The immunofluorescence results were consistent with the PCR results, suggesting that TSC2 KD microglia are activated (Figure 4B,C,E,F). In addition, we compared the ROS levels of the two groups. The results revealed that the ROS levels in the TSC2 KD group were significantly greater than those in the control group, suggesting that lowering the TSC2 levels promotes oxidative stress in microglia (Figure 4G,H).

### 2.7. Validation of Inflammasome-Related Hub Genes and NLRP3 Inflammasome Activation

**Figure 5:**
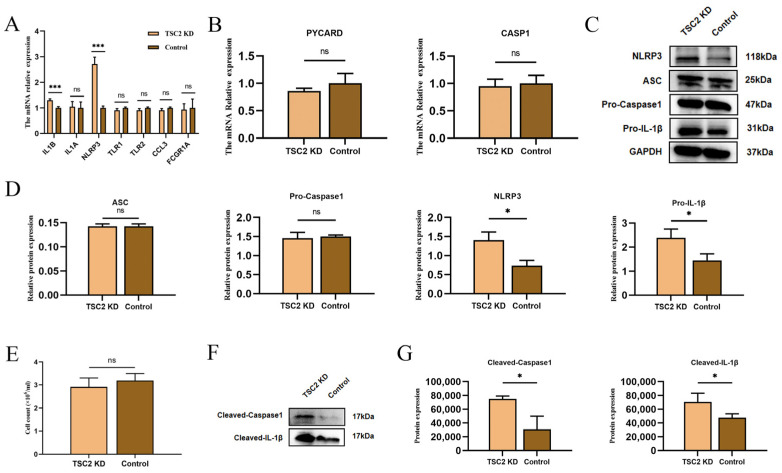
Comparison of inflammasome-related hub gene expression and NLRP3 inflammasome activation in HMC3 cells between the TSC2 KD group and the control group. (**A**) RT-qPCR results of seven inflammasome-related hub genes in the cells of the two groups revealed that the expressions of the NLRP3 and IL1B genes were elevated in the TSC2 KD group and that there were no significant differences in the expressions of the other five genes. (**B**) The expression levels of the PYCARD and CASP1 genes were not significantly different between the two groups. (**C**,**D**) NLRP3 and Pro-IL-1β protein levels were greater in the TSC2 KD group than in the control group, but ASC and Pro-Caspase 1 protein levels were not significantly different between the two groups. (**E**) There was no significant difference in cell counts used to extract the supernatant between the two groups. (**F**,**G**) Cleaved-Caspase 1 and Cleaved-IL-1β levels were increased in the supernatant of TSC2 KD microglia. N = 3; ns *p* > 0.05, * *p* < 0.05, *** *p* < 0.001.

We subsequently validated the expression of inflammasome-related hub genes by RT-qPCR. The results revealed that NLRP3 and IL1B expression was significantly greater in the TSC2 KD group than in the control group, while there was no significant difference in the expressions of the IL1A, TLR1, TLR2, CCL3, and FCGR1A genes (Figure 5A). ITGAM was not tested because it is barely expressed in HMC3 [26,27]. We also examined the CASP1 and PYCARD genes, and RT-qPCR revealed no significant increase in the expression of these genes (Figure 5B). Western blot analysis revealed that the changes in the protein levels of NLRP3, Pro-IL-1β, ASC, and Pro-Caspase 1 were consistent with the change in the mRNA levels (Figure 5C,D). On the basis of similar total cell counts, we examined the expression of Cleaved-Caspase 1 and Cleaved-IL-1β in supernatants (Figure 5E,F). The results revealed that the TSC2 KD group produced more Cleaved-Caspase 1 and Cleaved-IL-1β than the control group did (Figure 5G). These findings suggest that a reduction in TSC2 promotes NLRP3 inflammasome activation and the IL-1β-associated inflammatory response in microglia.

### 2.8. Rapamycin Intervention

**Figure 6:**
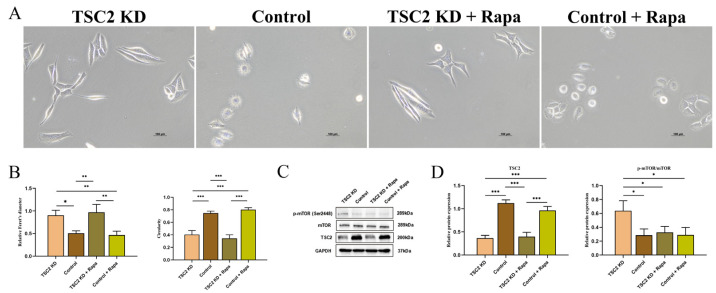
The effects of rapamycin intervention on the TSC2 KD model. (**A**,**B**) Feret’s diameter and circularity were similar for cells in the TSC2 KD and TSC2 KD + Rapa groups, whereas they were similar in the control and control + Rapa groups. (**C**,**D)** Microglia TSC2 protein levels were unchanged after rapamycin intervention, but mTOR phosphorylation was significantly reduced in the TSC2 KD + Rapa group. Rapa: rapamycin; N = 3; * *p* < 0.05, ** *p* < 0.01, *** *p* < 0.001.

We performed in vitro pharmacological interventions on HMC3 cells in the TSC2 KD group and the control group. Morphological analysis revealed that the rapamycin intervention had little effect on Feret’s diameter or circularity (Figure 6A,B). These findings suggested that the changes in cell morphology in the TSC2 KD group were not related to the phosphorylation of mTOR. As expected, the protein level of TSC2 in HMC3 cells remained unchanged following rapamycin intervention, but mTOR phosphorylation in the TSC2 KD + Rapa group was significantly inhibited (Figure 6C,D).

### 2.9. Effect of Rapamycin Intervention on Microglial Activation

**Figure 7:**
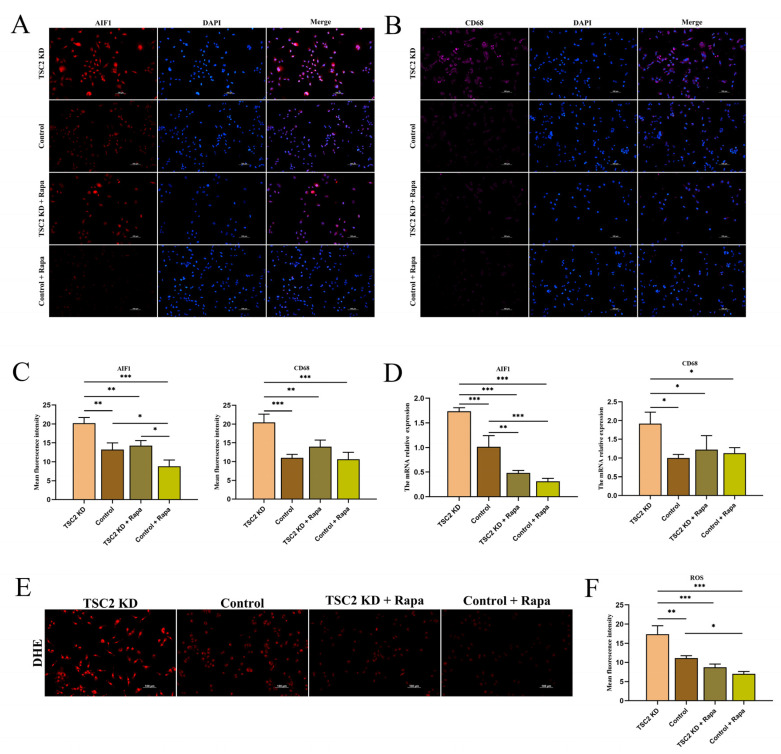
The effect of rapamycin intervention on HMC3 cells was detected. (**A**–**C**) Immunofluorescence assays revealed that AIF1 expression was downregulated in the TSC2 KD + Rapa group and the control + Rapa group and that CD68 expression was downregulated in the TSC2 KD + Rapa group. (**D**) RT-qPCR revealed a decrease in AIF1 mRNA levels in the TSC2 KD + Rapa group and the control + Rapa group and a decrease in CD68 mRNA levels in the TSC2 KD + Rapa group. (**E**,**F**) The level of ROS was significantly lower in the TSC2 KD + Rapa group than in the control + Rapa group. Rapa: rapamycin; N = 3; * *p* < 0.05, ** *p* < 0.01, *** *p* < 0.001.

The data revealed a significant decrease in AIF1 mRNA and a moderate decrease in fluorescence intensity in the TSC2 KD + Rapa group and the control + Rapa group (Figure 7A,C,D). After rapamycin intervention, the mRNA and fluorescence intensities of CD68 in the TSC2 KD + Rapa group decreased moderately, whereas the mRNA and fluorescence intensities of CD68 in the control + Rapa group did not change significantly (Figure 7B–D). The ROS levels were significantly lower in both the TSC2 KD + Rapa group and the control + Rapa group than in the TSC2 KD group and the control group (Figure 7E,F). These findings suggest that rapamycin intervention reduces the level of activation and oxidative stress in TSC2 KD microglia.

### 2.10. Effect of Rapamycin Intervention on the Activation of the NLRP3 Inflammasome in Microglia

**Figure 8:**
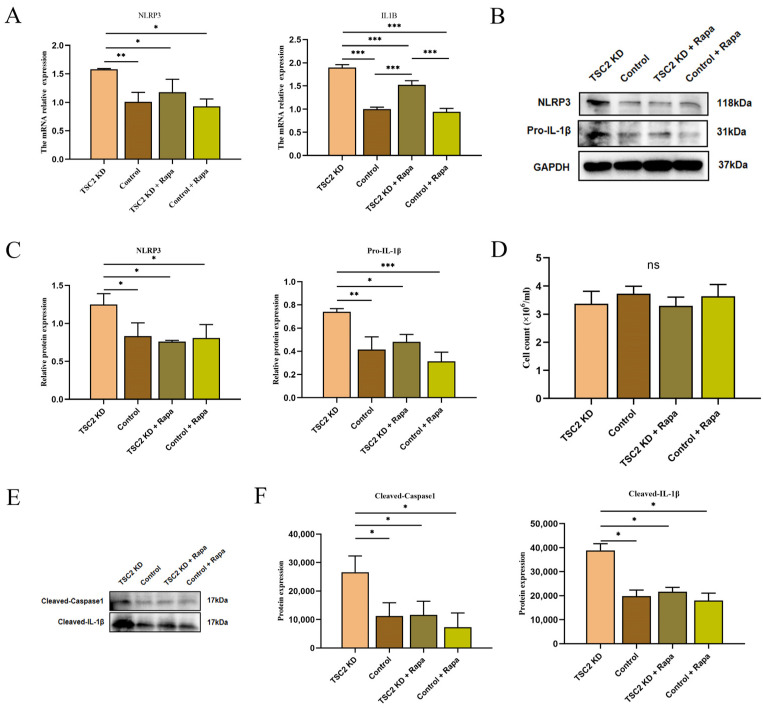
Detection of the effect of rapamycin intervention on NLRP3 inflammasome activation in TSC2 KD microglia. (**A**) RT-qPCR revealed that microglia in the TSC2 KD + Rapa group presented lower expressions of NLRP3 and IL1B than those in the TSC2 KD group. (**B**,**C**) Protein assays revealed that the levels of NLRP3 and Pro-IL-1β were lower in the TSC2 KD + Rapa group than in the TSC2 KD group. (**D**) There was no significant difference in the number of cells among the four groups used to extract the supernatant. (**E**,**F**) Cleaved-Caspase 1 and Cleaved-IL-1β levels were lower in the supernatants of TSC2 KD + Rapa microglia than in those of TSC2 KD microglia. Rapa: rapamycin; N = 3; ns *p* > 0.05, * *p* < 0.05, ** *p* < 0.01, *** *p* < 0.001.

The RT-qPCR analysis revealed that the transcription levels of the NLRP3 and IL1B genes in TSC2 KD cells were decreased following rapamycin intervention (Figure 8A). The protein-level analysis also revealed improvements in the levels of NLRP3 and Pro-IL-1β in TSC2 KD microglia after intervention (Figure 8B,C). Supernatant protein assays of different groups with similar cell counts revealed that Cleaved-Caspase 1 and Cleaved-IL-1β were significantly reduced in the TSC2 KD + Rapa group (Figure 8D–F). These data demonstrate that the activation of the NLRP3 inflammasome in HMC3 cells with TSC2 gene knockdown is associated with mTOR phosphorylation and that rapamycin intervention can inhibit these alterations.

## 3. Discussion

TSC can affect multiple organs, with neurological symptoms in childhood being the most common and severe. The cortical tuber is a characteristic feature of a disorder in late cortical development, comprising numerous giant cells, abnormal neurons, and activated glial cells. The study of cortical tubers has always been a focus of TSC research, and scholars have recently shown significant interest in the overactivated neuroinflammatory response within these tubers [14,16,28]. We utilized a publicly available transcriptome sequencing dataset of cortical tubers to investigate changes in the microglial inflammasome pathway in TSC patients. Initially, through bioinformatics analysis, we identified the upregulation of several inflammasome-related genes, including NLRP3 and IL1B, in cortical tubers. Our in vitro experiments confirmed that the downregulation of the TSC2 gene was correlated with microglial activation and the activation of the NLRP3 inflammasome. Moreover, we demonstrated that the phosphorylation of mTOR plays a role in NLRP3 inflammasome activation and that intervention with rapamycin reversed these effects. These findings suggest that TSC microglia participate in neuroinflammation by activating the NLRP3 inflammasome and that rapamycin exerts part of its therapeutic effect by regulating this process.

Although the pathological alterations in brain tissue in TSC are better understood, the biological mechanisms involved are still unclear. Abnormalities in glial cells are the etiology or significant pathogenic mechanism of various neurological disorders. The effects of astrocytes on TSC have received considerable attention, but the role of microglia has been reported in only a few studies to date. Unlike other brain-derived neuroglia, microglia originate from yolk sac primitive macrophages, migrate to the brain during embryonic development, affect brain development, maintain the neural environment, respond to injury, and repair damaged tissue [29,30]. Microglia are divided into resting and activated states, and activated microglia are involved in physiological and pathological functions such as removing cellular debris, repairing tissue, producing cytokines and chemokines, and coordinating with other nerve cells [29]. However, in disease states, microglial aggregation and persistent activation may adversely affect the brain. Previous studies have demonstrated an increased density of microglia and a large number of cells in an activated state from resected cortical tubers of TSC patients [13,31]. This aggregation is thought to be strongly associated with the epileptogenicity of cortical tubers [13].

Microglial activation has been observed in TSC surgical specimens, animal experiments, and molecular imaging studies [12,32,33]. Sun et al. [34] reported that the activation of microglia in the TSC may result in part from a decrease in the immunomodulatory molecules CD47 and CD200 on neurons. Astrocyte Tsc1 conditional knockout (CKO) also mediates microglial activation [7]. Nevertheless, sequencing data revealed the high expression of genes involved in the microglial activation pathway. Additionally, increased expression levels of AIF1 and CD68, as well as elevated ROS levels, in microglia with TSC2 knockdown further indicate that the cells are in an activated state, suggesting that the effects of TSC2 gene downregulation on microglia are autonomous. However, reversing microglial activation in astrocyte Tsc1 knockout (Tsc1^GFAP^CKO) mice was not effective in controlling seizures [7]. We speculate that the effects of cell-to-cell interactions on microglial function may be milder than those of direct TSC1/2 mutations in microglia. Studies have shown that defects in the Tsc2 gene can lead to abnormal primary microglial function during brain development and affect microglia-associated cell–cell contact-dependent physiological processes such as myelin formation [35]. In addition, in female TSC patients, the expressions of G-protein-coupled receptor 30, which plays important roles in neurodevelopment, neuroinflammation, and neuronal excitability, and its downstream protein kinase A are significantly downregulated in microglia [36]. Recent studies have revealed significant brain malformations, reduced synaptic density, and neuronal degeneration in microglial Tsc1 knockout (Tsc1^Cx3Cr1^CKO) mice, accompanied by severe epileptic seizures, suggesting that microglia may play an important role in TSC pathology [32,33]. Notably, the Tsc1^Cx3Cr1^CKO model may have some neuronal Tsc1 gene knockout and potentially may affect the interpretation of the role of microglia in this disease. Zhang et al. [32] induced 2-week-old Tsc1^Cx3cr1-CreER^CKO mice with tamoxifen and generated a model in which only 5% of Tsc1 knockout cells were non-microglia. The results revealed that none of the mice developed spontaneous seizures. However, in another study, four of the six TSC1^Cx3cr1-CreERT2^CKO mice induced with tamoxifen at 8–10 weeks developed spontaneous recurrent seizures, with an average of 1–1.5 seizures per day [33]. These inconsistent findings imply that more in-depth mechanistic explorations are needed to elucidate the role of microglia.

A growing body of evidence suggests that neuroinflammation is an important pathogenetic mechanism in neurological disorders [37,38]. As central nervous system macrophages, microglia mediate both innate and adaptive immune responses in the brain, such as responses to central nervous system infections and neurodegenerative diseases [39,40]. Activated microglia can be polarized into either pro-inflammatory (M1) or anti-inflammatory (M2) type, and excessive M1 polarization can lead to an inflammatory cascade and cause neurological damage. Pathological examination revealed that marked aggregates of activated microglia could be observed around aberrant neurons and giant cells in TSC patients [41]. The Tsc2 knockout model revealed a propensity for the M1 polarization of microglia, which further supports previous findings that microglia mediate TSC neuroinflammation [7,34,42]. A study of brain tissue from TSC children revealed that C1q complement protein expression was elevated in microglia and clustered near dendrites [6]. The origin of neuroinflammation in TSC is a debated issue, and both the activation of mTOR signaling and seizures are thought to lead to an inflammatory response. While seizures can induce the activation of glial cells and neuroinflammatory responses, previous research has revealed that inflammatory responses in astrocytes occur before seizure onset [10]. Although studies on whether TSC microglia independently generate an inflammatory response before seizures are lacking, the direct activation of BV2 microglia mTORC1 induces the expression of the pro-inflammatory factors TNF-α, IL-6, and HMGB1 and decreases the expression of the anti-inflammatory factors TGF-β and IL-10. Our in vitro studies also revealed that the mTOR signaling pathway is involved in the activation and inflammatory response of microglia but is independent of seizures and that regulating mTOR phosphorylation can alleviate these changes.

Previous studies have shown that IL-1β levels are elevated in TSC brain tissue and that astrocytes are an important source, but microglial IL-1β expression remains controversial [13,43]. Our study revealed that IL-1β production pathways were enriched and that IL-1β secretion was increased in TSC2 KD microglia. Overproduced IL-1β can act on astrocyte IL-1R1 receptors to promote the transcription of cytokines and danger signals associated with the nuclear factor kappa-light-chain-enhancer of the activated B-cell signaling pathway [8]. In addition, IL-1β is involved in inducing the transcription of cytokines such as IL-6, cyclooxygenase 2, and matrix metalloproteinases in TSC astrocytes, thereby exacerbating the neuroinflammatory response [8,9]. In this study, we demonstrated that NLRP3 inflammasome activation and Cleaved-IL-1β levels were elevated in microglia with TSC2 gene knockdown, indicating that microglia are involved in the development of neuroinflammation in TSC patients, which may lead to functional abnormalities in other neurons. The role of the NLRP3 inflammasome in neurological disorders has been confirmed by numerous studies [44,45,46]. The inhibition of NLRP3 inflammasome activation ameliorates epilepsy and neuropsychiatric disorders of multiple causes [47,48,49,50]. Therefore, interventions targeting the NLRP3 inflammasome may be promising research directions. Previous studies have shown that NLRP3 inhibitors alleviate neurologic injury, and in these studies, the application of rapamycin resulted in a similar effect [21,22,23]. mTORC1 downregulates autophagy by inhibiting multiple targets, and autophagy may inhibit the inflammatory response by suppressing the production of mitochondrial ROS, degrading assembled inflammasomes, and segregating Caspase 1 from Pro-IL-1β. When autophagy is impaired, the level of NLRP3 inflammasome activation can be significantly increased. Chen et al. [22] reported that the use of rapamycin reduced the levels of NLRP3, ASC, and Caspase 1 p20 fragments and the secretion of IL-1β in brain tissue. Our study also revealed that rapamycin intervention inhibited the activation of NLRP3 inflammasome in TSC2 KD microglia, which in turn reduced neuroinflammation. This finding reinforces the rationale for treating TSC patients with cortical tubers via mTOR inhibitors.

This study also has some limitations. Firstly, we were unable to determine whether other nervous systems were involved in NLRP3 inflammasome activation. Secondly, the indicators related to pyroptosis were not explored in this study. These limitations will be fully explored in our future research.

## 4. Materials and Methods

### 4.1. Downloading and Processing of the Transcriptome Dataset

**Figure 9:**
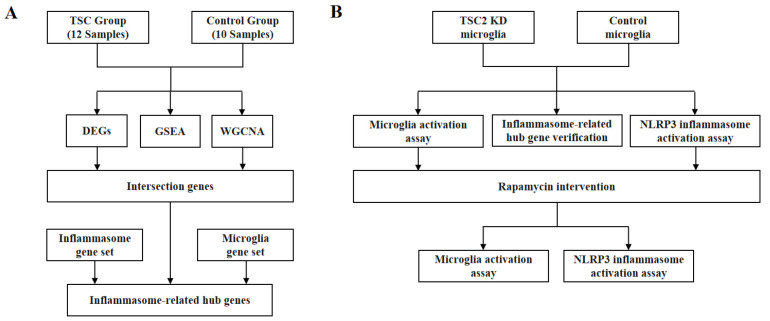
Flowcharts of (**A**) bioinformatics analysis and (**B**) cellular experiment.

The flowcharts illustrate the strategy of this study (Figure 9). The RNA-seq dataset (EGAS00001002485) from Mills et al., which is publicly available in the European Genome-Phenome Archive (https://ega-archive.org/datasets, 27 December 2022), was utilized, comprising 12 TSC cortical tuber samples and 10 cortical autopsy samples. Information on all enrolled patients has been reported in previous articles [51]. Data collection and analysis of the expression matrix were performed by R software 4.2. The ‘org.Hs.eg.db’ package was used to convert probe names to gene symbols, whereas the ‘AnnoProbe’ package was used to annotate gene product types and exclude nonprotein-coding genes. Genes with a total expression sum of less than 40 across all samples were excluded, and the expression matrices of the remaining genes were transformed using the log2(x + 1) function. The ‘limma’ package facilitated the differential analysis of expression matrices, with significance set at a *p* value < 0.05 and |log FC| > 1 as criteria to identify DEGs. A volcano diagram and a heatmap were generated using the DEGs and the |log FC| largest 50 genes among the DEGs, respectively.

### 4.2. Gene Set Enrichment Analysis

Gene set enrichment analysis (GSEA) identifies the enrichment location of specific gene sets within a dataset on the basis of expression levels, indicating the activation or inhibition of pathways in the subjects. In this study, GSEA was performed on all genes using the ‘clusterProfiler’ package version 4.4.3, and the enrichment results were further screened to explore pathway alterations related to microglial activation, neuroinflammatory responses, inflammasome complex formation, and IL-1β production. A *Q* value < 0.05, *p* value < 0.05, and |NES| > 1 were used as the criteria for GSEA.

### 4.3. Weighted Gene Co-Expression Network Analysis

WGCNA identifies and clusters highly correlated genes, and each clustered module can be associated with external clinical features to study core genes in the network. Data quality was judged to ensure that missing values and outliers were not present when the systematic clustering tree of the samples was plotted. The ‘WGCNA’ package version 1.72 was employed to construct a gene co-expression network and to select the soft threshold according to the guidelines of the scale-free structure. Different branches of the clustering tree and different colors represent different gene modules. The adjacency matrix was converted to a topological overlap matrix to mitigate the effects of spurious noise. The external trait relationships demonstrated module significance and were used to screen for the WGCNA gene set. The most relevant module was subsequently analyzed for correlations with clinical features.

### 4.4. Intersecting Gene Identification

DEGs and WGCNA gene sets were used for the identification of intersecting genes. Two gene sets, each containing 1932 microglia-associated genes (microglia gene set, Supplementary Table S1) and 920 inflammasome-associated genes (inflammasome gene set, Supplementary Table S2), were obtained from the GeneCards database (https://www.genecards.org/, accessed on 19 November 2023). Referring to these two gene sets, GAMNIs were identified from intersecting genes.

### 4.5. Construction of the Protein–Protein Interaction Network and Identification of Inflammasome-Related Hub Genes

The STRING database (https://cn.string-db.org/, accessed on 30 December 2023) was used for PPI network construction for the GAMNIs. The PPI network was visually represented using Cytoscape (version 3.9.0). Eight algorithms from cytoHubba (Stress, Betweenness, Radiality, Closeness, BottleNeck, EPC, Degree, and MCC) were employed to identify the top 20 genes. An UpSet plot was generated to display the results of inflammasome-related hub gene identification, and correlation analyses were conducted to elucidate gene correlations. To increase the confidence level, we applied t-tests to verify the mRNA expression of inflammasome-related hub genes, and *p* < 0.05 was considered significant.

### 4.6. Functional Enrichment Analysis

We utilized inflammasome-related hub genes for GO functional enrichment analysis, encompassing the MF, CC, and BP, using the ‘clusterProfiler’ package.

### 4.7. Microglial Culture and Construction of the TSC2 Gene Knockdown Model

The HMC3 was purchased from a company (Procell, Hubei, China). HMC3 cells were cultured in T25 culture flasks supplemented with growth medium (89% DMEM, 10% fetal bovine serum, and 1% penicillin and streptomycin). The cells were passaged when the density reached 90%, 1.5*10^5^ cells were inoculated per T25, and the growth medium containing the transfection reagent and lentivirus (Genechem, Shanghai, China) was replaced 24 h after cell inoculation. Compared to the TSC1 gene, patients with mutations in the TSC2 gene have a greater incidence and more severe symptoms of neurological comorbidities. Therefore, in this study, the TSC2 gene was knocked down to construct a model. The sequences of the lentivirus-transfected TSC2 siRNAs (TSC2 KD group) and scrambled siRNAs (control group) are shown in Table 1. The medium was changed to lentivirus-free growth medium 8 h after transfection, and the cell line stably transfected with lentivirus was screened with 2 μg/mL puromycin after 72 h. The fluorescence ratio of the cells was observed by fluorescence microscopy, and a stably transfected cell line was considered to be successfully constructed when all the cells expressed GFP fluorescence. RT-qPCR and Western blot experiments were used to characterize the expression of the TSC2 gene, and TSC2 expression was significantly reduced in the TSC2 KD group, which represented the completion of model construction.

### 4.8. Rapamycin Intervention

HMC3 cells in the TSC2 KD and control groups were treated with either 10 nM rapamycin (MedChemExpress, Monmouth Junction, NJ, USA) or an equivalent solvent when they reached 70–80% confluence and were then incubated for 24 h. After 24 h of treatment, the mRNA, protein lysate, and medium supernatant were extracted. In addition, rapamycin was added when the cell density reached approximately 10%, and the mixture was used for morphological analysis and immunofluorescence detection 24 h after the intervention.

### 4.9. Cell Viability Test

HMC3 cell suspensions from the TSC2 KD and control groups were prepared separately at a concentration of approximately 1 × 10^4^/mL in growth media. They were then seeded into 96-well plates with an inoculum volume of 100 µL per well, and media without cells served as a blank control. The cells were incubated at 37 °C in a 5% CO_2_ incubator for 23 h. Subsequently, 10 µL of CCK-8 reagent (MedChemExpress, Monmouth Junction, NJ, USA) was added to each well and incubated for 1 h in the incubator. The absorbance was measured using a full-wavelength microplate reader (Thermo Fisher Scientific, Waltham, MA, USA) with the detection wavelength set at 450 nm, and the average absorbances were calculated by subtracting the values of the blank control from the values of all the assays.

### 4.10. Cell Morphological Analysis

Bright-field images of the cells were captured using a light microscope (Nikon, Tokyo, Japan). Feret’s diameter and circularity, as measured by ImageJ software version 1.54, were used to assess the differences in cell morphology between the groups. Feret’s diameter was normalized on the basis of the values of the first sample in the KD group, whereas circularity was calculated as (4π*Area)/(Perimeter*Perimeter).

### 4.11. Real-Time Quantitative Polymerase Chain Reaction

Total mRNA was extracted by an adsorption column, and DNA removal and reverse transcription were performed according to the manufacturer’s instructions (Accurate Biology, Changsha, China). Each sample was heated at 95 °C for 30 s, then subjected to 40 cycles of 5 s at 95 °C and 30 s at 60 °C, and finally, a melt curve cycle of 10 s at 95 °C, 5 s at 65 °C, and 5 s at 95 °C was used. The expression of the target gene was compared to that of the housekeeping gene to determine the relative expression. The data were normalized beforehand, ensuring that the mean relative expression of all the mRNAs in the control group was set to 1. Table 2 summarizes the primers used in this study (Sangon, Shanghai, China).

### 4.12. Western Blot

In each well of a 6-well plate, equal densities of cells were seeded and cultured until they reached 90% confluence. Total protein lysates were collected using precooled protein extraction reagent and added to 5× protein loading buffer at a 4:1 ratio, followed by incubation at 100 °C for 5 min. For electrophoresis, different concentrations of SDS-PAGE were utilized for different proteins, with 20 μg of protein loaded into each lane. The proteins were subsequently transferred onto polyvinylidene fluoride membranes. Blocking was performed using 5% skim milk powder, followed by overnight incubation with the primary antibody at 4 °C and subsequent incubation with secondary antibodies at room temperature for 1 h. The primary antibodies used were as follows: TSC2 (rabbit polyclonal, 1:2000, Proteintech, Wuhan, China), NLRP3 (rabbit polyclonal, 1:500, Servicebio, Wuhan, China), Pro-Caspase 1 and Cleaved-Caspase 1 (rabbit polyclonal, 1:2000, Proteintech, Wuhan, China), ASC (mouse monoclonal, 1:500, Santa, Santa Cruz, CA, USA), Pro-IL-1β (rabbit polyclonal, 1:2000, Proteintech, Wuhan, China), Cleaved-IL-1β (rabbit polyclonal, 1:500, Abcam, Waltham, MA, USA), and GAPDH (mouse monoclonal, 1:20,000, Proteintech, Wuhan, China). A Super-Sensitive ECL Luminescence Reagent was used for antigen–antibody complex detection. The bands were visualized by electrochemiluminescence (Bio-Rad, Hercules, CA, USA).

To prepare the serum-free cell supernatant, approximately the same number of HMC3 cells was grown in each 6 cm dish, and the medium was changed to serum-free medium when the cell density reached 70%. The supernatant was collected when the cell density reached 90%, and the cells were digested and centrifuged to prepare a 1 mL cell suspension. The number of cells was measured using an automated cell counter (Logos Biosystems, Anyang-si, Republic of Korea), and the average value was taken by measuring the number of cells per plate twice. The supernatants from the serum-free cell culture medium were collected and concentrated to detect secreted proteins. Secreted proteins were layered with a 1/4 volume of chloroform and an equal volume of methanol, and the separated proteins were washed with an equal volume of methanol and centrifuged. The protein precipitates were dried at 55 °C for 5 min and dissolved in 60 µL of 2× protein loading buffer. The amount of protein loaded per lane was 10 µL, and the subsequent detection method was the same as that for the intracellular proteins.

### 4.13. Cell Immunofluorescence Staining

The cells were immersed in 4% paraformaldehyde at room temperature for 15 min and then washed with phosphate-buffered saline (PBS). The cells were subsequently treated with reagents containing 0.5% Triton X-100 and 5% fetal bovine serum for 30 min. Anti-AIF1 primary antibody (recombinant rabbit monoclonal, 1:500; HUABIO, Hangzhou, China) and anti-CD68 primary antibody (mouse monoclonal, 1:500; Proteintech, Wuhan, China) were incubated at 4 °C overnight. The next day, the residual primary antibody was removed by washing with PBS, and the cells were incubated for 2 h at room temperature with a goat anti-rabbit fluorescent secondary antibody (1:2500, Abcam, Waltham, MA, USA) or a goat anti-mouse fluorescent secondary antibody (1:1000, Abcam, Waltham, MA, USA). Nuclei were counterstained with DAPI staining solution (LEAGENE, Beijing, China) for 10 min at room temperature after washing with PBS. Finally, the cells were observed and images were acquired using a fluorescence microscope (Nikon, Tokyo, Japan).

### 4.14. Reactive Oxygen Species Detection

A reactive oxygen species fluorometric assay kit (Elabscience, Wuhan, China) was used to detect intracellular ROS levels in HMC3 cells. The cells were incubated with 10 μM working solution at 37 °C for 1 h. The cells were subsequently washed three times with PBS to remove the residual working solution and dried, after which images were captured with a fluorescence microscope (Nikon, Tokyo, Japan).

### 4.15. Statistical Analysis

All the data are presented as the means ± standard deviations. Differences between the two groups were compared using a *t*-test. Differences among multiple groups were assessed using one-way ANOVA, followed by pairwise comparisons between groups using the Tukey test. Statistical analysis was performed with SPSS 26.0, and *p* < 0.05 was considered statistically significant. Histograms were generated using GraphPad Prism 8.0.1.

## 5. Conclusions

In summary, we detected a high expression of genes related to the microglial activation and NLRP3 inflammasome pathways and identified eight microglial inflammasome-associated hub genes by analyzing a transcriptome sequencing dataset of cortical tubers from TSC patients. By constructing an in vitro TSC2 KD microglial model, we verified the conjectures on TSC microglial activation, NLRP3 inflammasome activation, and increased IL-1β secretion. It was also proven through rapamycin intervention that mTOR is the upstream molecule of these changes. These findings provide new data to support the neuroinflammatory argument for TSC. Further research is needed to explore the role of microglia in TSC neuroinflammation.

## Figures and Tables

**Table 1:** Control and TSC2 siRNA sequences.

NO.	5′	STEM	LOOP	STEM	3′
TSC2-RNAi (16136-1)-a	Ccgg	ACGAGTCAAACAAGCCAAT	CTCGAG	ATTGGCTTGTTTGACTCGT	TTTTTg
TSC2-RNAi (16136-1)-b	aattcaaaaa	ACGAGTCAAACAAGCCAAT	CTCGAG	ATTGGCTTGTTTGACTCGT	
GV112-NC-1	CCGG	TTCTCCGAACGTGTCACGT	TTCAAGAGA	ACGTGACACGTTCGGAGAA	TTTTTG
GV112-NC-2	AATTCAAAAA	TTCTCCGAACGTGTCACGT	TCTCTTGAA	ACGTGACACGTTCGGAGAA	

**Table 2:** Rt-qPCR primer sequences.

Primer Name		Sequence (5′ → 3′)
IL1B	Forward	GACCTGGACCTCTGCCCTCTG
	Reverse	GCCTGCCTGAAGCCCTTGC
IL1A	Forward	GACCAACCAGTGCTGCTGAAGG
	Reverse	GCCGTGAGTTTCCCAGAAGAAGAG
NLRP3	Forward	CCTGGTCTGCTGGATCGTGTG
	Reverse	CGGTGGTGGTCTTGGATGTCTG
AIF1	Forward	AGGATGATGCTGGGCAAGAGATC
	Reverse	TCAGGGCAACTCAGAGATAGCTTTC
TLR1	Forward	CAATGCTGCTGTTCAGCTCTTCT
	Reverse	GGTGCCCAATATGCCTTTGTTATCC
TLR2	Forward	CTACCAGATGCCTCCCTCTTACCC
	Reverse	TGCCACCAGCTTCCAAAGTCTTC
CCL3	Forward	TCTGCAACCAGTTCTCTGCATC
	Reverse	TGCTCGTCTCAAAGTAGTCAGC
FCGR1A	Forward	CTCCTTCTACATGGGCAGCAAGAC
	Reverse	GCAGCCTCGCACCAGTATAACC
TSC2	Forward	CAGACAATGGGAGACACATCACCTAC
	Reverse	CCAAGTTCACCAGCACCAGAAGG
CASP1	Forward	CACACCGCCCAGAGCACAAG
	Reverse	TCCCACAAATGCCTTCCCGAATAC
PYCARD	Forward	GCCCACCAACCCAAGCAAGATG
	Reverse	CTCCGCTCCAGGTCCTCCAC
GAPDH	Forward	GCACCGTCAAGGCTGAGAAC
	Reverse	TGGTGAAGACGCCAGTGGA
CD68	Forward	CTTCTCTCATTCCCCTATGGACA
	Reverse	GAAGGACACATTGTACTCCACC

## Data Availability

The EGAS00001002485 dataset can be downloaded after the request has been approved by submitting a request to the administrator of the European Genome-Phenome Archive (https://ega-archive.org/datasets). Experimental data will be made available on request.

## Supplementary Materials

The following supporting information can be downloaded at: https://www.mdpi.com/article/10.3390/ijms26157244/s1.

## Abbreviations

TSC	Tuberous sclerosis complex
IL-1β	Interleukin-1β
KD	Knockdown
ROS	Reactive oxygen species
mTOR	Mammalian target of rapamycin (mTOR)
NLRP3	NOD-like receptor protein 3
ASC	Apoptosis-associated speck-like protein containing a CARD
Pro-Caspase 1	Precursor cysteinyl aspartate-specific proteinase 1
CKO	Conditional knockout
Rapa	Rapamycin
DEGs	Differentially expressed genes
GSEA	Gene set enrichment analysis
NES	Normalized enrichment score
FC	Fold change
WGCNA	Weighted gene co-expression network analysis
GAMNIs	Genes associated with microglial NLRP3 inflammasome
PPI	Protein–protein interaction
GO	Gene Ontology
MF	Molecular Function
CC	Cellular Component
BP	Biological Process
HMC3	Human microglia cell line
RT-qPCR	Real-time quantitative polymerase chain reaction
PBS	Phosphate-buffered saline
DHE	Dihydroethidium
